# Placental Growth Factor (PlGF) in Women with Suspected Pre-Eclampsia Prior to 35 Weeks’ Gestation: A Budget Impact Analysis

**DOI:** 10.1371/journal.pone.0164276

**Published:** 2016-10-14

**Authors:** Suzy Duckworth, Lucy C. Chappell, Paul T. Seed, Lucy Mackillop, Andrew H. Shennan, Rachael Hunter

**Affiliations:** 1 Women's Health Academic Centre, King's College London, London, United Kingdom; 2 Oxford University Hospitals NHS Trust, Oxford, United Kingdom; 3 Research Department of Primary Care and Population Health, University College London, London, United Kingdom; Queen's University, CANADA

## Abstract

**Objective:**

To model the resource implications of placental growth factor (PlGF) testing in women with suspected pre-eclampsia prior to 35 weeks’ gestation as part of a management algorithm, compared with current practice.

**Methods:**

Data on resource use from 132 women with suspected pre-eclampsia prior to 35 weeks’ gestation, enrolled in a prospective observational cohort study evaluating PlGF measurement within antenatal assessment units within two UK consultant-led maternity units was extracted by case note review. A decision analytic model was developed using these data to establish the budget impact of managing women with suspected pre-eclampsia for two weeks from the date of PlGF testing, using a clinical management algorithm and reference cost tariffs. The main outcome measures of resource use (numbers of outpatient appointments, ultrasound investigations and hospital admissions) were correlated to final diagnosis and used to calculate comparative management regimes.

**Results:**

The mean cost saving associated with the PlGF test (in the PlGF plus management arm) was £35,087 (95% CI -£33,181 to -£36,992) per 1,000 women. This equated to a saving of £582 (95% CI -552 to -£613) per woman tested. In 94% of iterations, PlGF testing was associated with cost saving compared to current practice.

**Conclusions:**

This analysis suggests PlGF used as part of a clinical management algorithm in women presenting with suspected pre-eclampsia prior to 35 weeks’ gestation could provide cost savings by reducing unnecessary resource use. Introduction of PlGF testing could be used to direct appropriate resource allocation and overall would be cost saving.

## Introduction

Pre-eclampsia complicates 4–8% of pregnancies. [1] Diagnosis is time consuming and resource intensive. In women with suspected pre-eclampsia, current clinical management requires high-cost monitoring, fetal surveillance, [1] and medical management. This increases the likelihood of antenatal admission and possible iatrogenic preterm delivery. [2] In the US in 1992, $20 billion was spent on managing women with pre-eclampsia and their babies. [3]

Pre-eclampsia is unique to pregnancy and is characterised by an abnormal inflammatory and vascular response, resulting in increased vascular resistance, coagulopathy, endothelial dysfunction and subsequent poor placentation. Recent advances in understanding suggest placentally-derived angiogenic and anti-angiogenic factors [4] could be used to predict disease severity. [5–7] In normal pregnancy, placental growth factor (PlGF) concentrations increase with gestation, with concentrations peaking at 26–30 weeks [6] and declining towards term. PlGF concentrations are abnormally low in women with preeclampsia. [7]

We have recently conducted a prospective multicentre study, investigating the diagnostic accuracy of PlGF concentration in women presenting with signs and/or symptoms suggestive of preeclampsia in the second half of pregnancy. [7] The primary outcome was a diagnosis of preeclampsia requiring delivery within 14 days. Study findings identified PlGF as an important and reliable diagnostic tool in the management of suspected pre-eclampsia, in women below 35 weeks’ gestation. Test performance statistics revealed high sensitivity (0.96) and negative predictive value (0.98) for a diagnosis of pre-eclampsia. The introduction of PlGF testing could target those women at greatest risk for increased surveillance, whilst avoiding unnecessary intervention and resource use in those with subsequent normal outcomes.

This study provided us with an opportunity to supplement data from the literature with actual resource use to calculate the cost of current practice and model the savings if PlGF were used in management decisions. A treatment algorithm, (a diagrammatic depiction of potential management pathways), to be used alongside the PlGF test, allowed us to hypothesise how pregnant women might be managed based on PlGF and other clinical characteristics. The aim of this analysis was to evaluate the cost impact on local NHS budgets, using a decision analytic model, of introducing PlGF testing in this cohort of women if management were based on revealed PlGF results. We hypothesised that additional measurement of PlGF could aid clinical decision-making as to appropriate place of care and frequency of monitoring.

## Methods

### Participants

We undertook a prospective observational, cohort study investigating the role of PlGF testing in 625 women with suspected pre-eclampsia, between January 2011 and February 2012, in seven centres across the UK and Ireland. [7] Women were eligible for the study if they had signs and/or symptoms of suspected pre-eclampsia, were between 20^+0^ and 40^+6^ weeks of gestation with a singleton or twin pregnancy and were aged ≥16 years. Women with confirmed pre-eclampsia at the time of presentation were not eligible. Written informed consent was obtained and baseline demographic and pregnancy-specific information were entered onto the study database. As part of the budget impact analysis we conducted a detailed case note review of the resource use and pregnancy outcomes of 132 women enrolled in the cohort study prior to 35 weeks’ gestation from two sites (London and Oxford). A sample of women presenting prior to 35 weeks’ gestation was selected from those in a large inner city hospital (n = 109) and from a smaller site (n = 23) such that all 13 major diagnostic groups associated with hypertension and proteinuria were represented in the sub-set, together with all women with no hypertension, no proteinuria (protein: creatinine ratio <30mg/mmol) and with no diagnosis of pre-eclampsia prior to delivery. Random sampling from the resulting list was undertaken using a statistical program to produce a sub-group for detailed case note review. Retrospective review of paper case notes and electronic records relating to patient flow and attendance, together with imaging and laboratory testing was carried out to record health service usage, including outpatient appointments, day assessment attendance, hospital admissions and ultrasound surveillance during the two week period after their enrolment to the study. Participants gave informed consent and the study followed institutional guidelines.

Plasma samples were tested for PlGF using the Triage^®^ PlGF Test (Alere, San Diego, California) by trained laboratory staff at the UK site where the sample was taken. All participants had delivered and had pregnancy outcomes recorded before biomarker concentrations were analysed and revealed. Using a threshold cut-off of the 5^th^ centile, a PlGF concentration below this was classed as ‘low PlGF’. A PlGF concentration above 100pg/ml (equivalent to the 5^th^ centile) was classed as ‘normal PlGF’. A PlGF concentration below 12pg/ml was categorised as ‘very low PlGF’. Diagnoses of mild, moderate, and severe hypertension were made using criteria dictated by National Institute for Health and Care Excellence guidelines for the management of hypertension in pregnancy; [8] diagnosis of preeclampsia was made through adjudication by senior physicians using international definitions. [9]

### PlGF treatment algorithm

The National Institute for Health and Care Excellence guidelines on the management of hypertensive disorders in pregnancy advocate admission for all women diagnosed with pre-eclampsia, with severity of hypertension and fetal well-being directing management and timing of delivery; timing of delivery is dependent on maternal and fetal condition and neonatal intensive care availability. [8] This guideline was used to inform the ‘current treatment’ algorithm.

**Fig 1:**
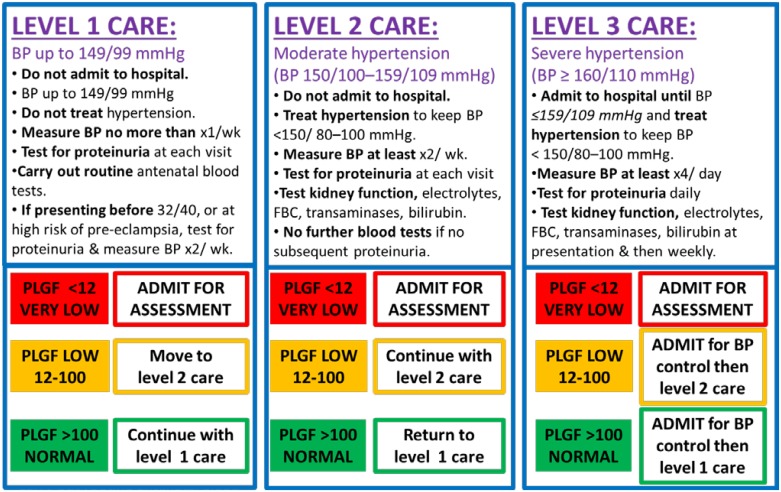
Clinical management algorithm: Use of PlGF at point of care in suspected pre-eclampsia.

Actual resource use, extracted from retrospective case note review, was applied to the treatment model, allowing theoretical comparison of economic burden. Fig 1 shows a clinical management pathway, based on data from our cohort study, that uses measurement of PlGF alongside blood pressure and proteinuria to risk stratify women with suspected pre-eclampsia.

### Decision analytic model

**Fig 2:**
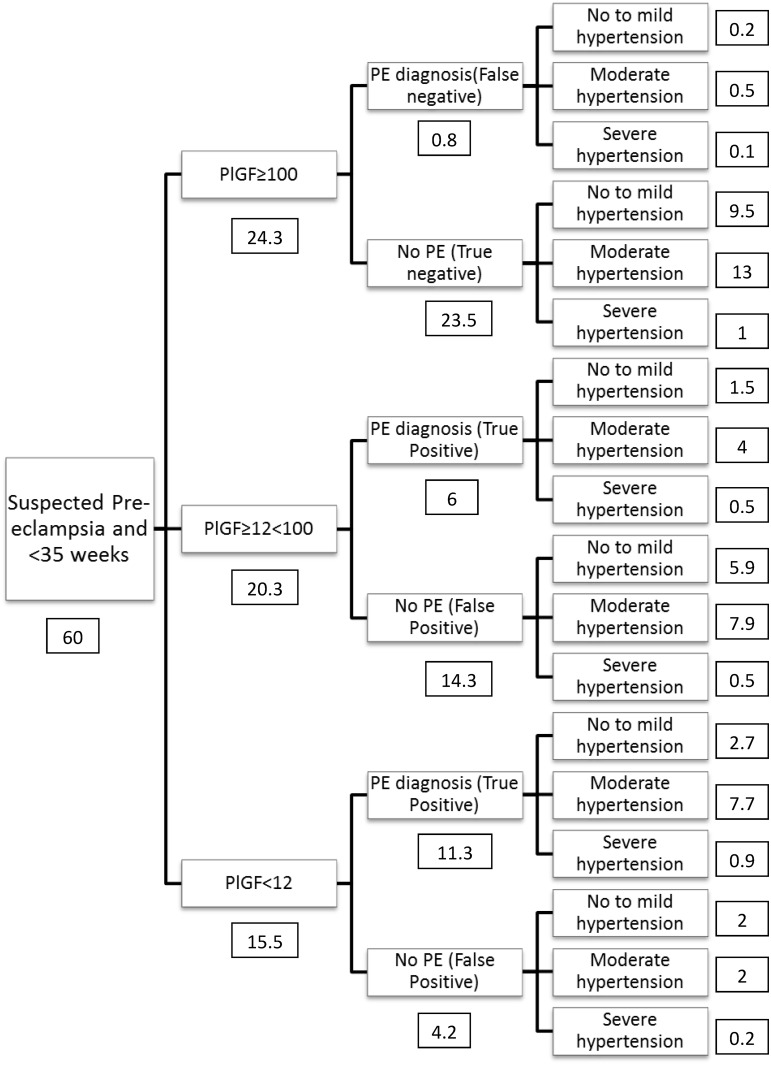
Flow diagram of algorithm using PlGF concentrations. Numbers shown in boxes relate to the numbers of women (from population of 1000 pregnant women) in the adjacent box.

A decision model was developed to assess the budget impact of introducing PlGF testing as a diagnostic adjunct compared with current practice. The model used a hypothetical cohort of 1,000 women who are assumed to have the same characteristics as 1,000 consecutive pregnant women presenting to an antenatal service in England. Costs for current treatment, without PlGF, are taken from women recruited as part of the prospective cohort study. The cost of PlGF plus management algorithm is calculated using a decision analytic model. Using the proportions derived from our study data (Fig 2), we calculated (i) the number of women who would be tested for pre-eclampsia using PlGF (ii) the number of women who fall into each of the three PlGF categories (iii) the number of women who would eventually have a diagnosis of pre-eclampsia or not in each of the resulting branches (iv) the number of women with no, mild to moderate or severe hypertension in each of the resulting branches. The parameters used to calculate the number of women in each branch are shown in Table 1. Of the 1,000 women, it is assumed that only women presenting with suspected pre-eclampsia undergo PlGF testing. Given that the treatment for women who do not present with suspected pre-eclampsia remains the same in both arms of the model their costs have not been included in the model.

**Table 1:** Presumed population parameters.

Diagnosis per 1000 women	Percentage (95% CI)	Source
Suspected pre-eclampsia	20% (10%-30%)	Clinical expert
Suspected pre-eclampsia <35 weeks	6% (4%-8%)	Clinical expert
**Disease Incidence**		
Incidence of pre-eclampsia	1.8% (0.8%-2.5%)*	Clinical expert
Percentage with moderate hypertension in women diagnosed with pre-eclampsia	68% (60%-76%)	Anumba et al (2010)
Percentage with severe hypertension in women diagnosed with pre-eclampsia	8% (4%-12%)	Anumba et al (2010)
Percentage with moderate hypertension in women without a diagnosis of pre-eclampsia	55% (50%-60%)	Anumba et al (2010)
Percentage with severe hypertension in women without a diagnosis of pre-eclampsia	4% (2%-6%)	Anumba et al (2010)
**PlGF test characteristics (<35 weeks predictive for the next two weeks)**		
Sensitivity PlGF>100pg/ml	96% (89%-99%)	Chappell et al (2013)
Specificity PlGF>100pg/ml	55% (48%-61%)	Chappell et al (2013)
Sensitivity PlGF<12pg/ml	63% (51%-74%)	Chappell et al (2013)
Specificity PlGF<12pg/ml	90% (85%-94%)	Chappell et al (2013)
Cost of PlGF test	£50	Alere

*There is no good estimate of the prevalence of pre-eclampsia in women <35 weeks’ gestation with estimations varying widely. We have used a conservative estimate at the lower end of the potential prevalence based on clinical opinion, as using a greater percentage increases cost-savings.

### Health care resource use

Health care resource use for the current treatment group presenting with suspected pre-eclampsia prior to 35 weeks’ gestation was calculated from women in the case note review. Women were divided by the three different PlGF test thresholds: <12 pg/ml PlGF; PlGF ≥12<100 pg/ml; or PlGF ≥100 pg/ml and into three different groups of hypertension: normotensive or mild hypertension; moderate hypertension; or severe hypertension for a total of nine groups. As clinicians in the study were not aware of the PlGF test result the resource use for each group represents current practice based on clinical impression only, with no knowledge of PlGF concentrations. It is assumed that on average women present at 31 weeks’ gestation for the PlGF test and that all women have 2 weeks of costs.

Resource use was evaluated by (i) percentage of women that accessed the service (ii) the mean number (and standard deviation) of times women accessed the service or average length of stay in the case of inpatient admissions (sub-divided into those that had fewer than five days length of stay and those with greater length of stay to reflect the different tariff payments for long and short stay women).

Health care resource use for the ‘PlGF plus management’ algorithm was based on the current treatment algorithm (Fig 1) and the National Institute for Health and Care Excellence Hypertension in Pregnancy Guideline. [8] Health care resource use was calculated in the same way as for the ‘current treatment’ arms, except that a weighted average was included for the proportion of women in each group with proteinuria (given that this would increase the likelihood of women being admitted). The proportion of women in each group with proteinuria was calculated from the 288 records in the cohort study where the baseline measurement of PlGF, proteinuria and blood pressure was taken prior to 35 weeks’ gestation (Table 2).

**Table 2:** Percentage of pregnant women with PCR>30 mg/mmol in the prospective cohort study.

Hypertension	Normotensive to mild	Moderate	Severe
PlGF≥100 pg/ml	26%	27%	29%
PlGF≥12<100 pg/ml	42%	30%	59%
PlGF<12 pg/ml	76%	64%	70%

The cost of routine diagnostic tests (such as serum transaminases, urinary protein estimation) and medication were not included as reliable recorded data were not readily available. Given that they represent a small percentage of the total cost of care the decision was taken to omit them rather than invest in the resources required to collate unreliable data: a blood test costs less than £2 per test and the most commonly prescribed medications, labetalol and nifedipine, cost between £0.05 and £0.50 per tablet. Additionally, these costs would be included in the tariff and hence would not represent an additional cost to the payer.

### Cost Perspective

The model is from the budget perspective of a commissioner, the organisation responsible for buying health care, within the National Health Service (NHS) in England. All costs are for the 2013/2014 financial year. Costs were obtained from 2013–2014 NHS tariffs and 2011–2012 reference costs (Table 3). Reference costs were converted to 2013–2014 values using the average last two years (2011/12 and 2012/2013) Hospital and Community Health Services price increase index. [10]

**Table 3:** Cost parameters.

	Cost per unit	Reference
Hospital admission—length of stay up to 5 days	£789	NHS PbR Tariff*[11]
Hospital admission—cost per day after 5 days	£377	NHS PbR Tariff*
Outpatient appointments	£284	NHS PbR Tariff*
Additional specialised ultrasound	£116	Reference costs[12]
Day unit cost (not admitted)	£378	NHS PbR Tariff*

* NHS PbR tariff: National Health Service Payment by Results tariff

#### Confidence intervals

Confidence intervals were calculated using Monte Carlo simulation for 1,000 iterations of the model to calculate the Monte Carlo error and associated 95% confidence intervals. The percentage of iterations where the model reported a cost saving are also reported. All percentages were modelled using a beta distribution and health care resource using a gamma distribution. Point estimates only were used for health care resource use associated with the treatment algorithm. The impact of different assumptions about health care resource use for the treatment algorithm on cost savings was tested as part of the deterministic sensitivity analysis. It was assumed that tariff, reference and PlGF costs were constant and hence these were also not varied.

#### Sensitivity Analysis

Studies have reported different point estimates for the incidence of pre-eclampsia and the presentation of risk factors indicative of pre-eclampsia in a pregnant population. We conducted two sensitivity analyses using the point estimates reported by Hadker et al (2010) [13] and Meads et al (2008). [14] The ‘PlGF plus management’ algorithm is based on guidelines for women presenting with suspected pre-eclampsia in whom there was additional information available on PlGF concentrations. There are no data directly available for actual resource use following implementation of the PlGF test and treatment algorithm as no trial has been conducted and formal implementation of the test has not been comprehensively reported. As a result we tested a range of best and worst case scenarios of health care resource use to assess the impact on potential cost savings from the PlGF test and treatment algorithm. The final price for PlGF test has not been confirmed. Additional analyses using the cost of £30 and £70 per test have been conducted.

### Materials and data availability

Documentation including study protocol and anonymised primary source data from the study can be made available to interested academic parties on request to the corresponding author. Health care resource use data were analysed using the statistical package Stata (version 11.2), College Station Texas, USA, and we used Excel 2010 to create the decision analytical model.

The study was approved by East London Research Ethics Committee (ref. 10/H0701/117) on 5 October 2010.

This manuscript has been written to adhere to CHEERS guidelines (S1 Table).

## Results

The resource cost (per 1,000 women) for two weeks following the PlGF test, according to diagnostic group, is summarised in Tables 4 and 5. Of 1,000 women, 60 presented with suspected pre-eclampsia prior to 35 weeks’ gestation and 18 (30%) had a final diagnosis of pre-eclampsia. In the model, one woman with a final diagnosis of pre-eclampsia had a PlGF concentration greater than 100 pg/ml (false negative). Nineteen women without pre-eclampsia had a PlGF concentration below 100 pg/ml PlGF threshold (false positives) and hence were managed using the PlGF algorithm even though they did not have a final diagnosis related to pre-eclampsia.

**Table 4:** Two-week costs of PlGF cost plus treatment algorithm compared to current practice for 1,000 pregnant women, based on correct identification of women with a final diagnosis of pre-eclampsia (deterministic).

PlGF (pg/ml)	Hypertension	Number of Women	PlGF + Algorithm Total Cost	Current Practice Total Cost	Difference
PlGF≥100	No/ mild hypertension	0.2	£139	£135	£4
	Moderate hypertension	0.5	£395	£576	-£181
	Severe hypertension	0.1	£103	£35	£68
**Total PlGF≥100**	**Total**	**0.7**	**£637**	**£747**	**-£110**
PlGF≥12<100	No/ mild hypertension	1.5	£1,314	£1,512	-£198
	Moderate hypertension	4	£5,623	£17,971	-£11,347
	Severe hypertension	0.5	£1,758	£1,099	£658
**Total PlGF≥12<100**	**Total**	**6**	**£8,695**	**£19,582**	**-£10,887**
PlGF<12	No/ mild hypertension	2.7	£2,825	£5,097	-£2,272
	Moderate hypertension	7.7	£20,681	£19,694	£986
	Severe hypertension	0.9	£4,401	£1,942	£2,459
**Total PlGF<12**	**Total**	**11.3**	**£27,907**	**£26,733**	**£1,173**
**TOTAL**		**18**	**£37,239**	**£47,061**	**-£9,823**

**Table 5:** Two-week costs of PlGF cost plus treatment algorithm compared to current practice for 1,000 pregnant women based on correct identification of women without a final diagnosis of pre-eclampsia (deterministic).

PlGF (pg/ml)	Hypertension	Number of Women	PlGF + Algorithm Total Cost	Current Practice Total Cost	Difference
PlGF≥100	No/ mild hypertension	9.5	£7,634	£6,847	£787
	Moderate hypertension	12.7	£10,240	£14,937	-£4,697
	Severe hypertension	0.9	£1,652	£565	£1,086
**Total PlGF≥100**	**Total**	**23**	**£19,525**	**£22,349**	**-£2,824**
PlGF≥12<100	No/ mild hypertension	6	£5,557	£6,394	£837
	Moderate hypertension	8	£11,265	£34,969	-£22,713
	Severe hypertension	1	£2,175	£1,360	£814
**Total PlGF≥12<100**	**Total**	**15**	**£18,988**	**£41,724**	**-£22,736**
PlGF<12	No/ mild hypertension	1.7	£1,787	£3,225	-£1,438
	Moderate hypertension	2.3	£6,195	£5,900	£295
	Severe hypertension	0.2	£815	£360	£455
**Total PlGF<12**	**Total**	**4.2**	**£8,798**	**£9,484**	**-£687**
**TOTAL**		**42**	**£47,311**	**£73,557**	**-£26,246**

The mean cost saving associated with the PlGF test (in the PlGF plus management arm) was £36,069 (95% CI -£, 99,307 to -£113) per 1,000 women. For each woman tested this equated to a cost saving of £635 (95% CI -£1454 to -£4). In 95% of iterations, PlGF testing was associated with cost saving compared to current practice. Hadker et al (2010) [13] used an incidence of pre-eclampsia of 4.0%, with 15% of pregnant women presenting with symptoms indicative of pre-eclampsia. If these figures are used in the model, holding all other variables at the baseline values, the mean cost saving per 1,000 women is £28,491 (95% CI -£106,836 to £12,508) with 81% of the iterations of the model demonstrating a cost saving. These assumptions produce a mean cost saving per woman, (with inclusion of the PlGF test), of £624 (95% CI -£2424 to £349), with the assumption that 45 pregnant women will present with suspected pre-eclampsia prior to 35 weeks’ gestation and hence PlGF concentrations will be measured.

If the incidence of pre-eclampsia reported by Meads et al [14] of 2.5% is used, the total cost-saving is £22,342 (95% CI -£93,516 to £4647) and 92% of iterations of the model are cost saving. Most cost savings were found in the moderate hypertension diagnostic group with a saving of £37,656 across 35 women. Women with a PlGF ≥12<100 pg/ml had a total cost saving of £33,623 (across 21 women). In the ‘current treatment’ group, 60% of women were admitted, 28% for longer than five days. This was high compared to women with no/ mild hypertension, where 39% were admitted, with 4% being admitted for fewer than five days (Table 6).

**Table 6:** Sensitivity analyses using the Monte Carlo Simulation model and 1,000 simulations.

Analysis	Cost of PlGF plus algorithm	Cost of current treatment	Difference	% Simulations PlGF plus algorithm cost saving
Algorithm admits all women with PlGF<100 pg/ml (assumes length of stay <5 days)	£106,261	£120,894	-£14,633	71%
Increase length of stay for all women admitted PlGF + algorithm by 3 days	£95,132	£120,894	-£25,761	81%
Algorithm admits all women with PCR> 30 mg/mmol	£95,182	£120,894	-£25,712	87%
Admission to inpatient ward costs 50% more	£92,403	£147,320	-£54,917	97%
Admission to inpatient ward costs 50% less	£78,089	£94,467	-£16,378	85%
PlGF test costs £30 per test	£84,046	£120,894	-£36,847	95%
PlGF test costs £70 per test	£86,446	£120,894	-£34,447	94%

## Discussion

The results of the decision analytic model suggest that, based on the best information available, there is a 95% chance that PlGF testing plus a treatment algorithm represents a cost saving for a commissioner’s budget compared to current practice. This cost saving is likely to be around £635 per woman over two weeks presenting prior to 35 weeks’ gestation with clinical characteristics indicative of pre-eclampsia or £36,069 per 1,000 pregnant women. These results are relatively robust to changes made to the assumptions in the model, although changes in the incidence of pre-eclampsia reduce the probability that PlGF plus management algorithm is cost-saving.

The main strength of this study is the comprehensive comparison of resource use in women undergoing PlGF testing for suspected pre-eclampsia. With most savings associated with pregnant women presenting with moderate hypertension, the ‘PlGF plus management’ algorithm potentially provides clinicians with the ability to stratify these women into risk groups more appropriately. Data were extracted from our recent prospective study, including participants encompassing a wide demographic and ethnic profile and a pragmatic approach to enrolment with minimal exclusion criteria, enabling generalisability. Final diagnoses were independently adjudicated by two senior clinicians following database record review, using strict criteria. PlGF concentrations were not revealed until all diagnoses had been adjudicated.

The model has a number of limitations. The findings produced have been simulated under certain assumptions, some derived from the prospective cohort study and others using nationally estimated cost parameters. These results are therefore hypothetical given a simulated scenario and are not from direct observation. If implemented into clinical practice, cost-savings may differ from the predictions of this model. PlGF has not yet been tested as part of a randomised controlled trial, meaning that there is uncertainty about what resource use pregnant women with suspected pre-eclampsia, tested with PlGF and managed using the treatment algorithm, would actually use. An improvement in health outcomes for women and their infants is not yet proven, although results of our study suggest that PlGF has the potential to aid diagnosis and assist decision-making, with subsequent impact on maternal and perinatal outcomes. Resource use may have varied costs in different settings, and so the cost savings presented here need to be reproduced in other settings.

The predictive diagnostic potential of PlGF testing is optimal below 35 weeks’ gestation, with outcomes reliably predicted in the two week period after testing (the primary outcome of our study). For the purposes of this analysis, therefore, we did not evaluate women presenting after 35 weeks’ gestation or assess resource use beyond the two week test period. It is now common practice to routinely deliver women with pre-eclampsia at 37 weeks. [15] This implies costs are likely to decline towards term, as hospital admission demands the greatest economic burden. [16] It was not possible to include additional diagnostic tests and therapeutic medications in the model, due to the lack of availability of this information. We believe, however, that this would produce a marginal change to the total costs and may well be captured as part of the tariff.

The results suggest that PlGF plus management algorithm presents a realistic and innovative adjunct to the management of women with suspected pre-eclampsia. The test was approved in May 2016 by the National Institute of Health and Care Excellence to be used in women presenting with suspected pre-eclampsia up to 35 weeks’ gestation alongside standard clinical assessment and subsequent clinical follow-up, to help rule-out pre-eclampsia (but not recommended yet for rule-in use until further research is available) [17]. The results are likely to be generalisable to other settings with similar maternity care, where women have regular antenatal care, with additional assessment as needed if a woman presents with suspected pre-eclampsia. The test performance of PlGF measurement has been reported to be similar across these settings, but costs of subsequent care may show some variation across different countries.

There has only been one previous report of health economic analysis using PlGF testing (in combination with soluble fms-like tyrosine kinase-1 at a fixed time—point of 20 weeks’ gestation as a screening test for development of pre-eclampsia later in pregnancy; Hadker and colleagues reported a decision analytical model using a hypothetical cohort of 1000 pregnant women and interviews with obstetricians, laboratory managers and healthcare payers to populate the model’s assumptions. They demonstrated that the costs of a typical pregnancy managed with the new test were £1781 per patient compared to £2726 with standard practice, with the cost savings resulting from better identification of true positives and negatives. [18] The weaknesses of this previous model include the use of test performance statistics from a case-control study (rather than a prospective cohort study as used here), lack of real-world health care resource use data and the use of non-validated data from physician and expert interviews for resource utilisation inputs.

In conclusion, PlGF testing is associated with improved predictive performance, in the diagnosis of preeclampsia, compared with current diagnostic practice in high risk women. It is likely that PlGF testing with linked treatment algorithm is cost-saving compared to current practice from the perspective of a health care commissioner over a two week period. Some uncertainties still remain that warrant further research with a prospective analysis of costs with actual implementation of PlGF.

## Supporting Information

S1 Table CHEERS Checklist. (DOC)
